# A Christmas tree cataract

**DOI:** 10.11604/pamj.2014.18.332.4780

**Published:** 2014-08-25

**Authors:** Fadoua Zahir, Hicham Tahri

**Affiliations:** 1 Ophthalmology Service, CHU Hassan II, Fez, Morocco

**Keywords:** Christmas tree, cataract, anterior nucleus

## Image in medicine

A 70-year-old man referred for cataract surgery, had on examination a palette of colored needle-shaped opacities in the cortex and anterior nucleus of the lens, consistent with unilateral Christmas tree cataract. This type of cataract result from the accelerated breakdown of membrane associated proteins. The peptides and amino-acids accumulate in the lumen of the reticular meshwork, and cystine is concentrated beyond the level of crystallization, giving rise to growing crystals.

**Figure 1:**
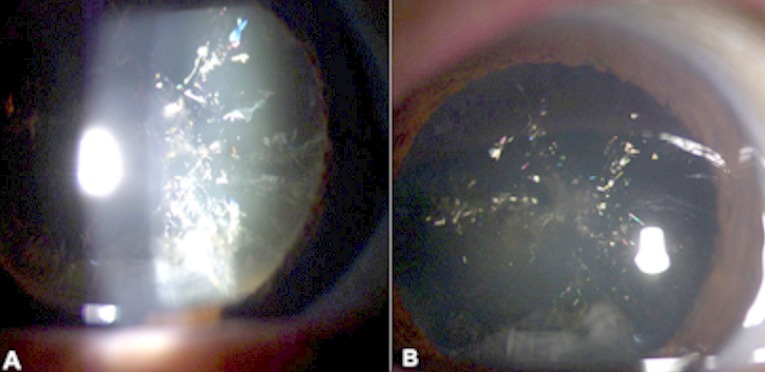
Palette of colored needle-shaped opacities in the cortex and anterior nucleus of the lens (A and B)

